# Which mechanisms explain the motivation of primary health workers? Insights from the realist evaluation of a maternal and child health programme in Nigeria

**DOI:** 10.1136/bmjgh-2020-002408

**Published:** 2020-08-25

**Authors:** Bassey Ebenso, Chinyere Mbachu, Enyi Etiaba, Reinhard Huss, Ana Manzano, Obinna Onwujekwe, Benjamin Uzochukwu, Nkoli Ezumah, Timothy Ensor, Joseph Paul Hicks, Tolib Mirzoev

**Affiliations:** 1 Nuffield Centre for International Health and Development, University of Leeds School of Medicine, Leeds, UK; 2 Health Policy Research Group, University of Nigeria Faculty of Medical Sciences, Nsukka, Enugu, Nigeria; 3 Leeds Institute of Health Sciences, University of Leeds, Leeds, UK; 4 Sociology & Social Policy, University of Leeds School of Sociology and Social Policy, Leeds, UK; 5 Faculty of Social Sciences, University of Nigeria, Nsukka, Enugu, Nigeria

**Keywords:** qualitative study, health systems evaluation, health policy, maternal health, child health

## Abstract

**Introduction:**

Well-trained, adequately skilled and motivated primary healthcare (PHC) workers are essential for attaining universal health coverage (UHC). While there is abundant literature on the drivers of workforce motivation, published knowledge on the mechanisms of motivation within different contexts is limited, particularly in resource-limited countries. This paper contributes to health workforce literature by reporting on how motivation works among PHC workers in a maternal and child health (MCH) programme in Nigeria.

**Methods:**

We adopted a realist evaluation design combining document review with 56 in-depth interviews of PHC workers, facility managers and policy-makers to assess the impact of the MCH programme in Anambra State, Nigeria. A realist process of theory development, testing and consolidation was used to understand how and under what circumstances the MCH programme impacted on workers’ motivation and which mechanisms explain how motivation works. We drew on Herzberg’s two-factor and Adam’s equity theories to unpack how context shapes worker motivation.

**Results:**

A complex and dynamic interaction between the MCH programme and organisational and wider contexts triggered five mechanisms which explain PHC worker motivation: (1) feeling supported, (2) feeling comfortable with work environment, (3) feeling valued, (4) morale and confidence to perform tasks and (5) companionship. Some mechanisms were mutually reinforcing while others operated in parallel. Other conditions that enabled worker motivation were organisational values of fairness, recognition of workers’ contributions and culture of task-sharing and teamwork.

**Conclusions:**

Policy designs and management strategies for improving workforce performance, particularly in resource-constrained settings should create working environments that foster feelings of being valued and supported while enabling workers to apply their knowledge and skills to improve healthcare delivery and promote UHC. Future research can test the explanatory framework generated by this study and explore differences in motivational mechanisms among different cadres of PHC workers to inform cadre-related motivational interventions.

**Key questions**

**What is already known?**

- It is widely recognised that the determinants of health worker motivation such as availability of material resources, salaries, training, supportive supervision are strongly context dependent.
- There is poor understanding of how motivation influences the behaviour of primary healthcare (PHC) workers in resource-constrained settings and what mix of intervention approaches are required to trigger motivation among PHC workers.

**What are the new findings?**

- Our paper suggests ways in which interventions can be grouped in a particular context to have an impact on motivation of salaried PHC workers and identifies specific mechanisms of how the interventions contribute to motivation.
- Five key mechanisms that explain the underlying mechanisms through which motivation works in the context of Nigeria are: (1) health workers feeling supported, (2) feeling comfortable, (3) feeling valued and recognised, (4) morale and confidence to perform tasks and (5) companionship.

**What do the new findings imply?**

- These findings underline the need for policy-makers and managers to implement a group of interventions to simultaneously address the multiple interrelated problems that constrain health worker motivation in low-income and middle-income countries.

## Introduction

Global interest in Universal Health Coverage (UHC) has endorsed the need for well-trained, adequately skilled and motivated primary healthcare (PHC) workers.^1^ Based on the WHO’s dimensions of well-performing workforce, motivated workers are more likely to be available, responsive to clients’ needs and deliver quality healthcare.^2^ Evidence also suggests that workforce motivation mediates how programme inputs (eg, supportive policies, resource availability, salaries and supervision) can contribute to staff performance.^3^ In other words, staff motivation interacts with factors in the work environment and wider social context to influence staff performance. While there is abundant literature on the determinants of staff motivation,^4–8^ the published knowledge on the mechanisms of how PHC worker motivation works is scarce, particularly from resource-constrained settings. Bhatnagar *et al*^3^ critique existing studies on the determinants of health workforce motivation for being descriptive in nature and for failing to explain the underlying mechanisms through which motivation works. They therefore recommended the use of theory-based research approaches to better understand the causal pathways of how motivation works. Our paper responds to this call.

This paper has three objectives. First, it contributes to the human resource for health literature through reporting results from a realist evaluation (RE; a theory-driven approach) which examined the mechanisms that explain how motivation works among PHC workers in a maternal and child health (MCH) programme in Nigeria. Second, it increases understanding of key contextual factors that enable or constrain PHC worker motivation in public healthcare facilities. Finally, it provides a theoretically based explanation of aspects of the MCH programme that impacted on workers motivation at individual, organisational and societal levels.

We start by defining work-related motivation, followed by the background of the MCH programme in Nigeria. The paper then describes the methodology adopted to assess motivation, and the results section describes the proposed causal pathways to PHC worker motivation in Anambra State, Nigeria. We conclude by discussing lessons for influencing policy and practice decisions for improving health workforce motivation in PHC settings.

### What is work-related motivation?

Work-related motivation is a contested concept with multiple definitions used in the literature. WHO defines worker motivation as the level of effort exerted by employees and their desire to perform well, and these are central determinants of quality of care.^2^ Most available research on health worker motivation in low-income and middle-income countries (LMICs) focus on motivation magnitude that activates work behaviour, and on the drivers of motivation.^9 10^ However, scholars like Pinder^11^ define work motivation to include a set of energetic forces that originate within and beyond individual workers to trigger work-related behaviour, and determine its form, direction, intensity and duration. Pinder’s definition suggests that in addition to motivational intensity, work motivation can be assessed by its origin and sustainability over time.^7 12^ This implies that motivation is a process that arises from interactions between individuals, their work environment (eg, organisational climate and leadership) and the broader context surrounding the work environment (eg, national funding practices and health reforms).^13^ To further Pinder’s perspective, researchers have developed several frameworks that divide work motivation theories into two broad categories: exogenous and endogenous theories.

Exogenous theories focus on explaining how contextual influences (ie, extrinsic factors) can be altered to improve or constrain work motivation, including how resource availability or its absence in the work place and wider social contexts influence motivation.^14^ Endogenous theories, on the other hand, use psychological mechanisms within individuals (ie, intrinsic factors) to explain work motivation through understanding how the satisfaction of human needs can boost employee motivation.^15^ Scholars further categorised intrinsic factors within individuals into two: (1) lower-level needs and goals that aid the satisfaction of basic survival needs such as shelter and personal safety, and (2) higher-level motives and goals that facilitate self-actualisation (eg, self-determination and sense of competence).^16^ Employees often take physiological needs into account in deciding about work space and lighting; about self-worth when they decide about recognition for work done; and self-actualisation when they decide about opportunities for challenging tasks.^13^ Included among exogenous theories are the goal theories, need theories, incentive/reward theories and reinforcement theory whereas the grouping of endogenous theories include equity theory, self-efficacy theory, intention theories and other cognitive theories.^17^

However, despite the growing interest in the drivers and mechanisms of work motivation, only three RE studies of mechanisms of workforce motivation were identified, two of which drew on self-determination theory to research motivation in autonomous community health volunteers in Uganda^18^ and in top-performing community health teams in El Salvador^19^ respectively. The third study combined the Herzberg’s two-factor theory of needs with the person-environment fit theory to identify mechanisms of employee turnover in Ethiopia.^20^ The methods and results sections of our paper draw on relevant endogenous and exogenous theories to explain how motivation worked among salaried PHC workers in Nigeria.

### The context of PHC in Anambra State of Nigeria

Anambra State, situated in southeast Nigeria, has a population of 4.45 million people. There are 1216 health facilities in the state, 34% of which (or 416 facilities) are government-owned while 66% (or 800 facilities) are faith-based institutions and privately owned facilities that mission hospitals, private hospitals and maternity homes. Three hundred and eighty-two of the 416 government-owned institutions (or 92%) are PHC facilities run by local government areas (LGAs), 32 of 416 institutions (or 7.7%) are secondary health facilities comprising general hospitals, comprehensive health centres and cottage hospitals managed by the state government; while the remaining two facilities (or 0.48%) are tertiary institutions managed by the state and the federal governments, respectively.^21^ The key categories of health workers at PHC level in the state include doctors, nurse/midwives, community health extension workers (CHEWs) and laboratory staff. There is paucity of data on actual numbers of health workforce and salaries of PHC workers in Anambra state. However, table 1 shows that the expected numbers of health workers per 1000 population in Anambra are lower than the national average.^22 23^ A baseline assessment of PHC facilities conducted in 20 103 found that although skilled antennal providers and birth attendants existed in Anambra state, they lacked drugs, basic tools and equipment to enable them provide high-quality maternity services.^24^ Most PHC facilities assessed were also dilapidated and needed renovation. The non-availability of material resources combined with the poor physical condition of PHC facilities to negatively affect infant and maternal mortality in Anambra state (see table 1). The above context provided justification for implementation of the Subsidy Reinvestment and Empowerment Programme (SURE-P) in Anambra State. The SURE-P programme is discussed next.

**Table 1:** Key PHC indicators in Anambra state (in 2013) compared to national average*

Primary healthcare indicators	Anambra state	National
Population density (people per km^2^)	992	442
Number of doctors per 1000 population	0.02	0.382
Number of nurse/midwives per 1000 population	0.5	1.026
Number of CHEWs per 1000 population	0.02	0.137
Women who received Antenatal care from skilled provider (%)	84	61
Proportion of births delivered in a health facility (%)	84.6	36
Proportion of births assisted by skilled personnel (%)	87.6	38
Women who attended postnatal care within 2 days of birth (%)	56	40
Infant mortality rate per 1000 live births	82	72
Maternal mortality ratio per 100 000 live births	1098	576

*Sources: Nigeria Demographic and Health Survey 2013,^22^ WHO health workforce statistics,^23^ Uzochulwu 2013.^24^

CHEW, community health extension worker; PHC, primary healthcare.

### The SURE-P MCH Programme

Between 2012 and 2015, the Government of Nigeria implemented a SURE-P in the Federal Capital Territory and 36 states of Nigeria, including Anambra State, to invest profits from fuel revenues into a social protection fund for vulnerable populations.^25^ The SURE-P had a MCH component (SURE-P/MCH) designed to ensure the good health of pregnant women and babies and consisted of supply and demand components. The supply component intended to increase access to high quality health services and improve MCH outcomes through: recruiting and training PHC workers (2000 midwives, 10 000 community health workers consisting of 1000 CHEWs and 9000 Village Health Workers), renovating health facilities and increasing availability of equipment, drugs and supplies.^25 26^ The demand component intended to increase pregnant women’s utilisation of health services for antenatal care (ANC) and childbirth using a conditional cash transfer (CCT) scheme as a resource. CCTs were given to those who registered at designated PHC facilities, received four ANC check-ups, delivered at participating health facilities and took their infants to receive the first series of vaccinations.^27^ In Anambra, the SURE-P/MCH programme deployed 12 new PHC workers (four midwives, two CHEWs and six village health workers) to complement already existing staff at each participating PHC facility.^28^

## Methods

### Study design

We used a RE approach to understand how motivation worked among PHC workers in an MCH programme, as well as to understand which contextual factors enabled or constrained PHC worker motivation in public healthcare facilities. RE, a theory-driven evaluation approach that builds, tests, validates and refines theories^29^ was used to understand the impact of the multi-intervention SURE-P/MCH programme on PHC worker motivation by clarifying ‘how and why the programme worked, for whom, in which circumstances and for how long’.^30^ Data collection was through document review and qualitative interviews with purposefully selected stakeholders. Context, mechanism and outcome (CMO) configurations were used as a heuristic tool to develop eight initial programme theories (IPTs) about how the MCH programme was intended to function in the context of Nigeria (see online supplementary material 1), and to inform the broader middle-range theories from the study. The process of developing the eight IPTs have been reported elsewhere.^27^ In this paper, we focus specifically on the IPT for workforce motivation. Next, we clarify the meanings of CMOs, and CMO configurations as used in this study.

10.1136/bmjgh-2020-002408.supp1 Supplementary data

According to Pawson and Tilley,^30^ it is not programmes that work, rather, it is the resources offered by programmes that enable stakeholders (eg, implementers and service users) to make them work. Mechanisms are the ways in which programme resources or strategies interact with the reasoning of stakeholders to produce effects^31^ and they can only be activated in certain circumstances, that is, in specific contexts. Context describes the features of the conditions in which programmes are implemented that trigger the mechanisms to produce intended and unintended outcomes. In this sense context can be categorised by level: micro (related to individual), meso (related to interpersonal) and macro (related to existing policies, economic conditions in Nigeria, organisational practices and cultural norms). Outcome patterns are the proximal, intermediate or distal effects of programmes that result from activation of different mechanisms in specific contextual circumstances.^30^ Through CMO configurations, the proposed effectiveness of a programme is outlined, with proposed explanation(s) of: (1) why programme outcomes turned out as they did, and (2) how the programme responded to underlying mechanisms and in what contexts.

### Data collection and analysis

REs are method neutral, often drawing on local effectiveness (quantitative) data to identify outcomes and on qualitative insights for theory generation, refinement and consolidation.^25^ To assess the impact of the MCH programme on worker motivation in Anambra State, Nigeria, we used a combination of document review and semi-structured qualitative realist interviews^32^ with 18 facility-based PHC workers, 16 facility managers, 12 policy-makers and 10 programme managers. Each interview lasted 45–60 min. Participants were recruited between January 2016 when phase 1 interviews were conducted as part of theory generation and June 2018 when phase 2 interviews were conducted to test theories generated in phase 1 (see table 2 for participants recruited during each phase). Interviews were conducted by female doctors (EE and CM) and a sociologist (NE) who were trained in realist interviewing techniques and the RE approach. Research staff provided study information sheets to potential participants to help them decide whether to participate in the study, giving them at least 72 hours to express an interest in being part of the study. Purposive sampling was used to ensure that all four groups (PHC workers, facility heads, policy-makers and programme managers) were represented in interviews. Interview guides were pre-tested before they were administered on the field. All interviews were audio recorded, transcribed verbatim and analysed manually. Documents reviewed included health policies, the SURE-P/MCH programme handbook and the national health management information system policy identified through discussions with programme managers and examined to ascertain the overall programme architecture and key assumptions. These informed the logic model which underpinned our inquiry^27^ and informed the IPTs including the one on health worker motivation reported here.

**Table 2:** Features of and methods adopted for data collection during the phases of study

Phase of study	Feature of phase	Method of data collection
Phase 1	Developed eight working theories and a logic model of how SURE-P/MCH is supposed to function. One of the eight theories sought to explain motivation of PHC workers.	Review of SURE-P/MCH programme handbook. Literature review of supply and demand sides of community health worker programmes. Interviews with 48 stakeholders. Health workers (n=13). Facility managers (n=13). Policy-makers* (n=12). Programme managers* (n=10). Stakeholder workshops with researchers (n=11).
Phase 2	Tested and refined health workers motivation theory.	Qualitative interviews with eight stakeholders: Health workers (n=5). Facility managers (n=3).
Phase 3	Verified and consolidated motivation theory. Elaborated the mechanisms of motivation.	Used Herzberg’s two-factor theory and Adam’s equity theory to verify mechanisms of motivation. Reasons for selecting these theories are explained shortly. Developed CMO configurations using data from phase 2 transcripts outlining how interactions between resources and reasoning operated at micro, meso and macro levels.

*Policy-makers and programme managers were interviewed at LGA, state and national levels.

CMO, context, mechanism and outcome; LGA, local government area; MCH, maternal and child health; PHC, primary healthcare; SURE-P, Subsidy Reinvestment and Empowerment Programme.

All data were analysed using a realist logic of analysis to make sense of, test and refine programme theories.^32^ During data collection and analysis, four data coders (EE, CM, NE and a research assistant) moved iteratively between analysis of particular examples, refinement of programme theory and application of abstract theory.^33 34^ Table 2 shows the features of data collection and analysis methods adopted in each phase of the study.

IPTs were developed during phase 1 of the RE through extracting tacit theories about what works and why from: (1) documents reviewed above; (2) a focused literature review of interventions on community health worker programmes; (3) one-on-one interviews with PHC workers, facility managers, policy-makers and programme managers; and (4) technical workshop with researchers.^27^ Tacit theories from documents and literature review informed the development of phase one interview guide (that was structured by the resources and interventions provided by SURE-P programme), to aid the exploration of how SURE-P resources and interventions led to outputs and outcomes that were depicted in the logic model. Findings of phase one interviews in turn informed the: (1) development of eight IPTs including the IPT for workforce motivation, and (2) identification of diverse factors at micro, meso and macro levels that triggered workforce motivation. These factors are summarised in the results section.

During phase 2 conducted in June 2018, IPTs were tested and refined iteratively (retroductively) during interviews with PHC workers and facility managers. A different interview guide was developed to aid theory. This interview guide (See online supplementary material 2) was structured by sections, with each section representing one of eight IPTs we were aiming to test. This paper presents findings from workforce motivation theory only. Each section of the interview guide started with narrative propositions of the theories, followed by specific questions which probed different aspects of the theory. In phase 3, the tested theories were consolidated using emerging data from transcripts of qualitative interviews. To facilitate the process of refining and consolidating best-fit programme theories for the MCH programme, we developed bespoke analytical configurations to visualise causal linkages between and among possible **C**ontexts, **M**echanisms and **O**utcomes within CMO configurations of theories. The CMO analytical configurations helped to outline how interaction between resources and reasoning can operate at micro, meso and macro levels.

10.1136/bmjgh-2020-002408.supp2 Supplementary data

We then drew on the Herzberg’s two-factor theory and Adam’s equity theory to explain how motivation worked during SURE-P, using supporting data from CMO analytical configurations. These two theoretical frameworks were selected after a scoping review of theories that supported the understanding of work motivation, which led to an initial shortlist of 11 prominent theories of motivation.^8^ This was followed by appraisal of the extent to which the shortlisted theories offered guidance for articulating how contextual factors at micro, meso and macro levels influenced PHC worker motivation. Finally, we selected the Herzberg’s two-factor theory and the equity theory as best-fit theories for explaining how staff motivation worked among PHC workers in Nigeria, prompted by emerging insights from interview transcripts highlighting the importance of resource availability in the workplace and of fairness of staff treatment for meeting the needs of health workers. We explain both theories next.

### Herzberg’s two-factor theory

Herzberg’s two-factor theory considers motivational factors that lead to job ‘satisfaction’ (eg, educational opportunities, sense of achievement, intrinsic interest in the work and involvement in decision making) to be distinct from hygiene factors that cause job ‘dissatisfaction’ when they are absent (eg, salary, good working conditions, recruitment policies and administrative practices).^35 36^ Herzberg named the de-motivators hygiene factors, as such factors are common in the work environment. According to the two-factor theory (see summary in table 3), motivational factors can be intrinsic or extrinsic to the individual whereas factors linked to job dissatisfaction (ie, hygiene factors) are contextual factors that are extrinsic to the individual. The principle of the theory is that improving motivation factors increases job satisfaction whereas the presence of hygiene factors decreases job dissatisfaction.

**Table 3:** Herzberg’s two-factor theory showing key components* of motivational and hygiene factors

Job dissatisfaction is influenced by absence of hygiene factors	Job satisfaction is influenced by presence of motivation factors
Working conditions Relationship with co-workers National/organisational policies, rules and culture Quality of supervision or leadership Base wage, salary Security	Achievement Recognition Responsibility Interesting work Advancement or promotion Personal growth

*This list and categories are not intended to be exhaustive.

### Adam’s equity theory

This theory focuses on a persons’ perception of fairness as a motivator.^37^ It states that employees are more likely to be motivated when they believe they are fairly treated, with such motivation triggering positive work attitude and behaviours. On the other hand, workers who feel unfairly treated are predisposed to being dissatisfied and display negative work attitudes and behaviour which manifest as increased absenteeism and low commitment.^38^ Adam’s theory introduced the idea of social comparison wherein motivation is based on what employees consider to be fair when compared with others. In this sense, employees assess organisational fairness by comparing, for example, their own remuneration and/or recognition of performance with those of their peers. According to this theory, contextual factors that influence employees’ perception of organisational justice include availability of resources (human and material), development opportunities and leadership style.

## Results

We report findings following the Realist And Meta‐narrative Evidence Syntheses Evolving Standards II guidelines for REs^39^ which recommend, in line with a realist approach, that existing theory is mixed with the developed programme theory to enhance the explanatory endeavour of the study.

The programme theory developed from testing and verification of IPTs during this study is:

‘In the context of human and material resource shortages, the SURE-P/MCH programme deploys adequate numbers of skilled workers, drugs and equipment and decent housing whilst ensuring regular remuneration, training, supervision and recognition for good performance. These inputs/resources generate a feeling of support, self-worth, empowerment and sense of camaraderie among PHC workers, leading to positive work behaviour and improved service delivery.’

The findings of the retroductive analysis of phase 1 interview transcripts revealed that a complex interplay of individual, organisational and wider social factors affected PHC worker motivation during programme implementation in Anambra State. Individual-level (intrinsic) motivation factors were workers’ interest in their vocation and concern for the welfare of patients. This supports other studies’ findings showing that altruistic behaviour among health workers are triggered by a desire to provide a good quality service to users’^40^ and to the communities they served.^19^ In our evaluation, seven organisational (extrinsic) drivers of worker motivation were: (1) increased availability and adequacy of material resources, (2) mentorship, (3) on-the-job training and supportive supervision, (4) regular payment of salaries, (5) recognition for good performance,^38^ (6) adequacy and good staff mix, and (7) renovation of facilities.^41–43^ Societal-level motivators included community appreciation for and recognition of workers’ roles. While analysis of findings of phase 1 identified the above seven drivers of PHC worker motivation, it did not explain how these factors generated health worker motivation.

Drawing on Gilmore *et al*’s work on data analysis and synthesis within a RE,^44^ we integrated the drivers of motivation from findings of phase 1 with emerging data from CMO analytical configurations developed from phase 2 transcripts and synthesised them to unravel the relationship between various drivers (or resources provided by SURE-P programme) and workforce motivation. The integration of findings from multiple data sources (and from different phases of our study) led to the identification of five significant explanatory patterns (ie, hidden causal forces) through which motivation worked in this programme: (1) feeling supported, (2) feeling comfortable with work environment, (3) feeling valued, (4) morale and confidence to perform tasks and (5) companionship. The five explanatory patterns or mechanisms are discussed shortly, beginning with narrative propositions, crafted as sub-theories of the consolidated programme theory above, and informed by linkages between/among CMOs and illustrated with supporting quotes from our qualitative data.

### Characteristics of stakeholders Interviewed

A total of 56 stakeholders were interviewed over the two phases of data collection for this study, 31 of whom (ie, 55.4% of stakeholders) were interviewed at LGA level; 13 people (or 23.2% of stakeholders) were interviewed at state while the remaining 12 people (or 21.4% of stakeholders) were interviewed at national level. Furthermore, 34 of the 56 people interviewed (or 61% of stakeholders) worked at PHC facilities as midwives, CHEWs or facility managers while the remaining 22 interviewees were policy-makers and programme managers at the state or national level. Twenty-seven of the 34 workers at PHC facilities (or 79.4%) were employed as permanent staff by the state or LGA while the remaining seven interviewees (or 20.6%) were employed on fixed-term contracts linked to delivery of SURE-P activities in Anambra State of Nigeria. See table 4 for details.

**Table 4:** Characteristics of respondents by stakeholder group and phase of data collection

Respondent group	Phase 1 interviews			Phase 2 interviews			Total
	LGA level	State level	National level	LGA level	State level	National level	
Midwives and CHEWs	13	0	0	5	0	0	18
Facility managers	10	3	0	3	0	0	16
Policy-makers	0	5	7	0	0	0	12
Programme managers	0	5	5	0	0	0	10
**Total**	**23**	**13**	**12**	**8**	**0**	**0**	**56**

CHEW, community health extension worker; LGA, local government area.

In addition to the characteristics shown in table 4, the description and analysis of the five mechanisms of motivation below will reflect on relevant differences or similarities in experiences of respondents to understand who benefited or not and how from SURE-P interventions. Although interviews and analysis did not explore different motivational factors for different professional groups (doctors, CHEWs, nurses/midwives), in general motivational factors affected groups similarly. However, facility managers appeared to be motivated by responsibility over other staff and by recognition (see table 3) for improved utilisation of services in PHC facilities, while midwives and CHEWs were motivated by availability of material resources, supportive work environment and access to training. Analysis further suggested that motivation depended less on professional grouping of staff but more on whether they were permanent or contract staff. These nuances are discussed next.

### Explaining mechanisms of PHC worker motivation

#### Supporting PHC Staff

In a context where health workers enjoy cordial working relationships and mentorship from senior colleagues, the provision of equipment and constant supply of drugs and consumables to PHCs increases PHC workers’ feeling of being supported because they have the necessary tools to work. The following quote from a CHEW illustrates how this mechanism was often explained by staff interviewed:

During SURE-P there were drugs and equipment. They also used to supply drugs and mama kits to the health facility…This made me feel better and happy because when our clients come, we had drugs to give to them. They [availability of resources] really motivated me to work and put more effort into caring for our clients because I had all it takes to work and give out those services…I was more motivated during SURE-P because those things that we needed to work were available but now [after the end of SURE-P] we don't have them again. (**Female Community Health Extension Worker**)

In the context of Nigeria, where lack of basic work tools is common,^10 45^ the availability of resources (drugs, equipment and delivery kit) at PHC facilities stimulates health workers to provide quality MCH services whereas resource shortages can cause dissatisfaction and reduced performance. While CHEWs generally felt supported by the availability of drugs and equipment, there were a few instances where facility managers did not feel supported by the availability of material resources alone. The quote below highlights one such context:

Some equipment that are needed have been provided and they are there. They are in this facility, although in some cases, there may not be anyone to manage [operate] them. Like in the laboratory department, I have equipment in the laboratory, but there is no technician to manage it, so there are some gaps there (**Female, Facility Manager**).

This suggests the facility manager are likely to feel more supported when availability of material resources is accompanied by the right mix of human resources. This indicates how what constitutes support may be different across different cadres of PHC staff in the same health facility depending on whether and how their professional needs were met by the programme activities. Next, we explore the impact of physical working environment on motivation.

#### Physical, functional and psychological comfort

Prior to implementing the SURE-P programme, many PHC facilities were rundown, lacking staff accommodation or supply of water and electricity. Renovating health facilities and providing staff accommodation within facility premises (when combined with availability of material and human resources discussed in the previous section) created a positive working environment that made staff comfortable and enthusiastic to work:

SURE-P gave us all the things we needed such as light [i.e. electricity], water and the other things too. When these things are provided the nurses are happy [satisfied] doing their work, no matter the little amount [i.e. low salary] they are getting, because our job is to save lives, whether you eat or you don't eat, you will try to put more effort to save lives [i.e. a sense of duty]. (**Female, Midwife**).

This mechanism relates to the workplace built environment framework^46^ that relates optimal staff performance to physical, functional (because it enables workers to do their tasks) and psychological comfort in workspace environments. The physical condition of the workplace (eg, refurbished facilities and availability of running water and electricity) prevents dissatisfaction and enables PHC workers to achieve their clinical goals of improving healthcare outcomes.

#### Feeling valued

Where PHC workers are underpaid and their efforts remain unacknowledged, regular payment of salaries and recognition of staff who perform well increases morale and commitment to work.

The SURE-P programme appeared to ensure regular payment of salaries of PHC workers, which triggered mechanisms of satisfaction and commitment to work. In explaining the benefits derived from the SURE-P programme, a CHEW stated:

I benefitted from the SURE-P programme in many ways. [The] first is that I was committed to my work during SURE-P programme. I was working happily because the payment [salary] we received at the time helped [sustained my commitment]. (**Female Community Health Extension Worker, contract staff paid by the SURE-P programme**)

While the above CHEW described regular payment of salaries as an indication of being valued by the health system, on the other hand, a facility manager felt valued by the responsibility to attract pregnant women to deliver in a health facility instead of in the community, although this responsibility was rewarded (or acknowledged) through financial incentives. Asked whether she was motivated by anything else apart from availability of material resources and adequacy of human resources, the facility manager said:

Yes of course, because when they came to pay the conditional cash transfer to the mothers who delivered here [at the heath facility], they also paid us 5000 Naira (13USD) as incentive… Not every staff, just myself as facility manager…[for managing] Village Health Workers who went into the communities to sensitize the pregnant mothers so that they will be coming here instead of going to the Traditional Birth Attendants in the community (**Female Facility Manager, permanent staff**).

Many health workers explained that salaries were paid promptly during the SURE-P programme, yet some permanent staff and employed by LGAs complained that the salary scale for paying workers in Anambra state was lower than at national level where salaries were paid by the Federal Government of Nigeria (see the Adam’s equity theory section). They cited disparity in salary scales as a cause of worker dissatisfaction. Similarly, a few permanent health workers employed by the LGA also reported that for some unknown reason, salaries were either delayed or unpaid by government after SURE-P ended. The next quote explains how nonpayment of salaries after SURE-P creates dissatisfaction:

Non-payment of salaries after SURE-P really affects it [i.e. work effort] because when staff are demoralized they won't come to work when they are supposed to come… [W]hen you come to the health facility you won't see them because they don’t feel appreciated… [T]hey will tell you that they have not been paid for the work they have done, and that there are no drugs [in the facility] for them to work with (**Female, Community Health Extension Worker, permanent staff**)

Here the non-payment of salaries by government generated feelings of being undervalued by the health system, leading to diminished organisational loyalty manifesting as absenteeism and non-delivery of service. Nevertheless, some PHC workers interviewed reported that community support for and roles recognition helped to sustain motivation when salaries were unpaid.

Taken together, the preceding subsections demonstrate how the combination of: (1) availability of material resources (drugs and consumables), (2) physical and psychological comfort and (3) regular payment of salaries prevented dissatisfaction through making PHC workers feel supported by the health system and their host community, whereas recognition for additional responsibility led to enhanced satisfaction to increase work effort in Nigeria.

#### Improving staff morale and self-confidence

In a context of irregular supervision and reduced prospects for professional training, the provision of supportive supervision and equitable opportunities for training to improve staff knowledge and skills make staff feel more confident to provide services.

We feel happy when we have regular training and supervision. The reason is that during SURE-P programme they used to train us for like 5 days every so often, [and] then we will step-down the training to other PHC staff. It is very necessary that, as a health professional, you update yourself with ongoing changes and things in the profession, or else you go out for continuous study. Regular training boosts one’s morale [self-worth] and motivates one. After going for those trainings you'd come back with new knowledge that you will put into the work, and things [health service] improve. (**Female Community Health Extension Worker, contract staff**)

However, not all PHC workers enjoyed regular training opportunities, as reported by a facility manager:

I didn’t benefit anything from SURE-P: no regular training, nothing, though there was a time we were called to Abuja for two to 3 days’ workshop – that’s all. But other staff working with me [i.e. SURE-P deployed staff] did benefit. They were paid [for attending trainings]. But we, the local government staff, we didn’t benefit anything. (**Female Facility Manager, permanent staff**)

While the SURE-P policy aimed to promote a culture of equal access to training, the last quote suggests that, in practice, only workers deployed by the SURE-P programme enjoyed retraining opportunities. As already highlighted in the background section of this paper, the SURE-P programme deployed 12 new health staff to participating facilities (comprising four midwives, two CHEWs and six village health workers) to complement already existing staff at each participating PHC facility.^28^ Prioritising newly posted staff for training (ie, organisational inequity) seemed to cause feelings of inequality and tension between facility managers (who were permanent staff) employed by the LGA and SURE-P deployed staff (who were contracted fixed-term to help implement SURE-P activities in the health facilities).

#### Camaraderie and shared workload

In Nigeria, given a chronic shortage and mal-distribution of PHC workers, deploying sufficient numbers and right skill mix of PHC workers to underserved areas generates a sense of camaraderie and shared workload during shifts which enables health workers to spend quality time in service provision for clients:

The way I feel is the way everybody [i.e. PHC workers] feels. When you have many staff in the facility, there will be division of labor and work will be smooth and easy. When you are working with somebody, you become friends with that person. Among the permanent staff, I was the only midwife as it is now, but when they were here there were four other SURE-P workers and I felt better. We used to discuss, you know, work was flowing. (**Female Midwife, permanent staff**)

Good relationships with co-workers in a health facility with adequate numbers of staff prevents dissatisfaction among PHC workers in Anambra State. It is not surprising therefore that, the abrupt reduction in staff numbers in another facility following the withdrawal of programme funding led to increased work stress:

The staff strength in this facility is very poor now but during the SURE-P programme, I had 21 staff under me (4 midwives, 2 CHEWs, 6 village health workers from SURE-P and 9 nurses [perhaps from the LGA]), but as of now, I have only one staff…Although we are managing but it is stressful on us…. Because the workforce has been reduced so low, it is affecting me and the other health workers. We are almost working round the clock. (**Female Facility Manager, permanent staff**)

Despite the manager’s attempt to manage increasing workload, the stress of working round the clock is beginning to constrain health workers’ motivation. Most workers interviewed emphasised the significance of interaction, peer-support and convenient working hours as important functional factors that enabled them to do their work effectively, communicate and connect with other professionals.

Apart from the five mechanisms explained above, our analysis identified four contextual conditions at micro, meso and macro levels that enabled workforce motivation to occur: a sense of duty to care for patients (individual level), the values of fairness and a culture of task-sharing and team work (organisational level), and recognition of workers’ contribution to improve the health and well-being of the local community (organisational and societal levels).

## Discussion

The programme theory examined in this paper focused on understanding how motivation works, while also explaining why and in what circumstances the SURE-P/MCH programme had an impact on workforce motivation. Using Herzberg’s two-factor and Adam’s equity theories to guide data analysis and synthesis led to development of a middle range theory in the form of five mechanisms that plausibly explain how PHC workers’ motivation occurred. The distinctive feature of this study is its identification of the intervention approaches necessary to prevent dissatisfaction and improve motivation among salaried PHC workers and the specific mechanisms that explain how the interventions contribute to motivation.

**Figure 1:**
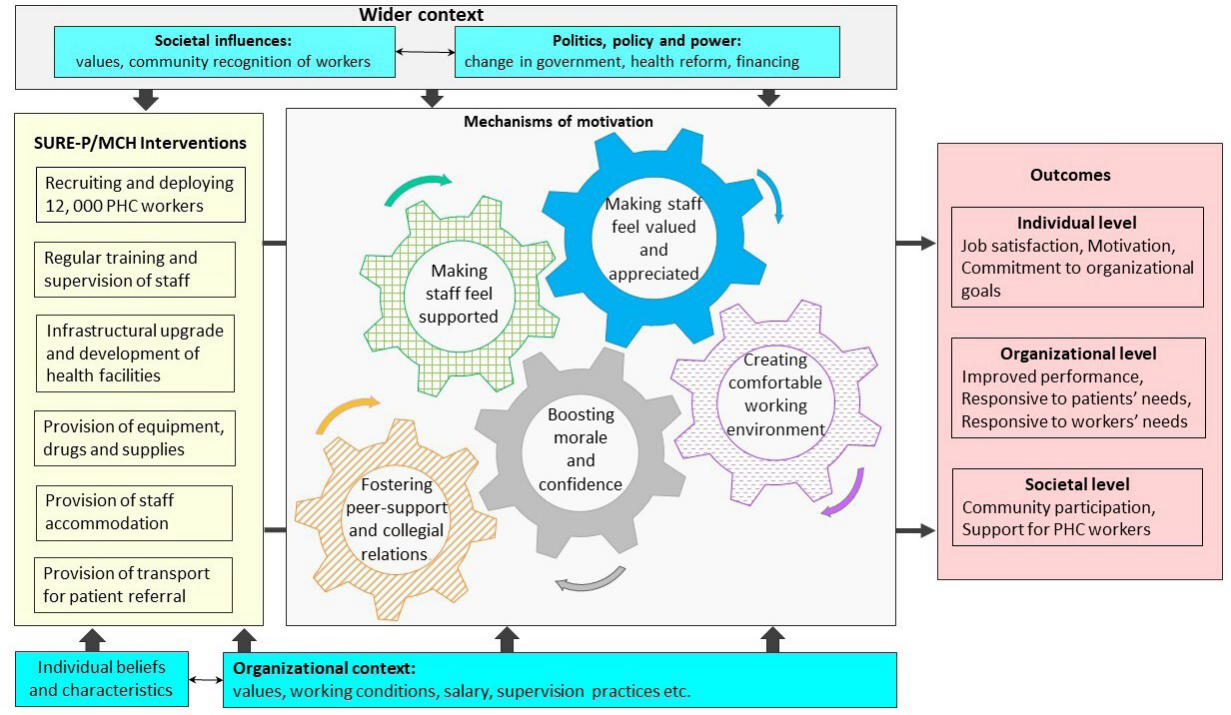
Conceptual representation of the ways in which SURE-P impacts PHC worker motivation.

The findings suggest that interventions of SURE-P interacted with the wider national and local context and an institutional environment generated by health sector reform policy, to boost motivation through: (a) making workers feel supported, (b) feeling valued, (c) creating comfortable working environments, (d) boosting morale and confidence and (e) fostering peer-support and collegiate relationships (depicted in the figure 1 below). The Figure shows the complex relationship between components of the theory highlighting how increased motivation is achieved when the ‘cogs’ of theory are in action. The figure provides an explanatory framework that accounts for organisational and wider contextual factors (coloured sky blue) that interact with programme interventions (see left hand column, coloured yellow) to improve/hinder motivation (see right hand column of outcomes, coloured red). Only individual-level outcomes are reported in this paper.

### Conceptual representation of the ways in which SURE-P impacts PHC worker motivation

Our data and the information in the left-hand column of the figure 1 identified five broad categories of interventions implemented by the SURE-P programme in Nigeria: (1) deploying appropriate skill-mix of workers in PHC facilities, (2) regular training and supervision of staff to maintain their knowledge and competence, (3) favourable working environment via infrastructural upgrade and staff accommodation, (4) provision of material resources to facilitate service delivery (ie, drugs, supplies and transport for referrals) and (5) financial compensation (salaries) and non-financial recognition for work done.

The overall findings of our research support the results of other studies of health worker motivation in Nigeria and other LMICs, which found that opportunities for professional development, optimal physical working conditions, strong supportive networks and a sense of being valued and appreciated by the health system and community motivate health workers to perform their jobs well.^7 12 47–49^ These findings have implications for developing strategies that ensure supportive supervision and strengthening community ties, establishing a fair recognition and reward system, and providing opportunities for training and career enhancement for health staff. While many studies focused on highly performing workers and institutions, or on specific cadres of workers (eg, community health volunteers) to identify what motivations and mechanisms drive good performance,^18 42 50–53^ our study assessed how motivation worked among different cadres of PHC workers (nurses, midwives and CHEWs) in an institutional environment influenced by changing health sector reform policy.

Similar to studies in Burkina Faso and South Africa^41 42^ we found that provision of equipment, drugs and consumables by SURE-P programme and workers perception of supportive supervision and mentoring were vital for making workers feel supported. Increased motivation during SURE-P was built on already-existing motivation of PHC workers generated by regular payment of salaries in Anambra state, Nigeria although salaries were sometimes lower than expected, delayed or unpaid. Regular remuneration triggered feelings of being valued by the health system, whereas perceptions of disparity in salary scales between state- and federal-owned facilities undermined motivation. Like studies in sub-Saharan Africa, PHC workers’ sense of being valued was reinforced when community members appreciated them for work done.^42 48^ This interplay between motivational factors suggest that, in the context of Anambra State, the mechanisms that trigger feelings of being supported and feelings of being valued or appreciated are mutually reinforcing as they enabled PHC workers to fulfil their professional goals.

In addition to regular remuneration and availability of material resources, our study found that the physical condition of the workplace was related to motivation. This has also been reported by previous studies, for example, in Ethiopia.^20^ We observed that refurbished infrastructural facilities complete with water and electricity supply increased functional capacity to perform tasks and PHC workers’ comfort, passion and enthusiasm for delivering MCH care. Another factor that improved service delivery was access to training opportunities. Fairness in implementation of training policies was also important to workers in Anambra state, who saw (re)training as a pathway to achieving personal growth and recognition for good performance.^42 51^ This also suggests that the causal mechanisms through which physical environments and access to (re)training influence worker motivation may be mutually reinforcing as both mechanisms act through increasing morale, psychological well-being (comfort) and enthusiasm to deliver services.

Besides impacting individual-level motivation we found that availability of material and human resources also sparked team-level motivation. Both individual and team-level motivations have been reported in El Salvador.^19^ We noticed that the SURE-P policy of deploying adequate numbers and the right skill-mix of PHC workers to health facilities increased team-level motivation through increasing peer-support and companionship among multi-cadre staff and reducing individual workload. By contrast, abrupt reduction in staff numbers following withdrawal of programme funding undermined team-level motivation through increasing workload and stress levels of the remaining staff, who were expected to work unsociable and long hours. We believe PHC workers in Nigeria may associate the abrupt reduction of staff numbers in health facilities to the sometimes unpredictable deployment and transfer decisions of policy-makers and health managers.^54^ Abimbola *et al*^54^ identified three broad mechanisms that underlie routine deployment and transfer decisions in Nigeria: (1) to enhance PHC worker experience, (2) deploying or posting PHC workers to improve service delivery in receiving health facilities and (3) deploying or posting PHC workers in response to requests from powerful actors. The SURE-P programme’s policy of recruiting and deploying PHC workers appears to support the second of Abimbola *et al*’s mechanisms. To our knowledge, this is the first RE in LMICs to consider the effect of cutbacks and withdrawal of programme funding on health worker motivation.

### Practical implications beyond Nigeria

While we acknowledge that diverse contextual factors (cultural, political and socioeconomic) influence workforce performance and that a one-size-fits-all approach will not address workforce performance issues in all contexts,^53^ nevertheless, our findings demonstrate the need for managers and policy-makers to implement a group of interventions to simultaneously address multiple interrelated problems that constrain workforce motivation and performance.^9 55 56^ In LMICs where health systems challenges continually impede the attainment of UHC, the mix of interventions can include approaches that involve: (1) deploying appropriate skill-mix of workers to address human resources shortages, (2) providing staff accommodation to address essential needs of workers, (3) improving the working environment infrastructurally and by supplying material resources to enable service delivery and (4) creating opportunities for regular skills training and supervision in ways that promote organisational justice.

### Study strengths and limitations

The key strength of our study is the identification of a mix of interventions implemented simultaneously by the SURE-P programme to stimulate access to quality MCH services. This mix of intervention approaches acted at multiple levels to improve job satisfaction and worker motivation through addressing competency-related deficiencies (individual level), staff shortages/attrition (organisational level) and undervaluing of PHC worker roles (health systems and societal levels).

There are three limitations of the study. First, although it identified patterns of motivational mechanisms of PHC workers at individual and interpersonal team levels, this paper excludes a comparison of factors that motivated different cadres of PHC workers in multi-disciplinary teams. This would have provided insight into similarity and differences in mechanisms of motivation among different cadres of workers. Second, this RE drew insight from health workers in Anambra state only. Including workers from other states of Nigeria may have identified additional mechanisms of motivation to further enrich our findings. Third, our analysis is based on staff self-reported data drawn from interviews conducted after the withdrawal of the SURE-P programme. The time lag between the end of SURE-P programme and when PHC staff were interviewed could have affected the recall of their experiences. As this study adopted a qualitative research approach, we did not include psychological surveys of interactions between different mechanisms of motivation nor of potential hierarchies among the mechanism identified and their combined effects on staff performance.

## Conclusions

The programme theory developed by our study identified causal pathways that plausibly explain how motivation of salaried PHC workers can be increased and sustained to contribute to health system improvements. The findings increase understanding around the potential for wider context and institutional structures and practices to enhance or inhibit workforce motivation. The study can also inform policy design in Nigeria and LMICs with similar contexts for creating positive working environments that foster a feeling of being valued and supported and enables PHC workers to use their clinical knowledge and skills to improve universal healthcare delivery. Future realist research should further this knowledge by testing the explanatory framework generated by this study, and explore differences in motivational mechanisms between different cadres of workers to inform cadre-related strategies for motivating multidisciplinary teams.
