# Supplementary material for: Which mechanisms explain the motivation of primary health workers? Insights from the realist evaluation of a maternal and child health programme in Nigeria
Source: BMJ Glob Health. 2020 Aug 25;5(8):e002408. doi: 10.1136/bmjgh-2020-002408 (PMC7449364; doi:10.1136/bmjgh-2020-002408)
Supplement: Supplementary data [file bmjgh-2020-002408supp001.pdf]

Supplementary material 1: 8 IWTs of the MCH programme in Nigeria

BOX 1: Supply side programme theories.

INITIAL WORKING THEORY (IWT1): contained in Phase 1 Methods Handbook

In the context of irregular payment of salaries and poorly functioning facilities in Anambra state **(C),** if different incentives (e.g. regular payments, training and improved working environment) are provided in a timely manner, then these interventions will make health workers feel motivated **(M)**, and lead to sustained performance, job satisfaction and improved retention of staff **(O).**

APPROVED PROGRAMME THEORIES—SUPPLY SIDE

PPT1: Prioritisation of MCH

In the context of poor health outcomes, interest from policymakers and politicians in maternal and child health care (MCH), combined with advocacy and lobbying from key policy actors to prioritise MCH **(C)**, is likely to help generate and maintain political and economic commitment across all tiers of government manifesting as a culture of ownership of MCH programs **(M)**, eventually leading to timely release of counterpart funds and availability of other resources (e.g. human, supplies), ultimately contributing to sustained implementation of and access to MCH services for vulnerable groups **(O)**.

PPT2: Improved staff motivation

In the context of staff shortages and lack of material resources, if adequate numbers and mix of skilled health workers are recruited, deployed to health facilities (that have security men, comfortable accommodation, regular electricity and water supply and transportation for emergency referrals); and if health workers receive adequate equipment, supplies and consumables for their work and are regularly trained, supervised and rewarded for good performance **(C)**, then health staff will feel motivated (i.e. appreciated and happy) to increase and maintain their performance **(M)** which is likely to lead to increased provision and utilisation of quality MCH services, ultimately contributing to improved service and health outcomes **(O)**.

PPT3: Health workers feeling safer to work

In the context where health facilities or communities ensure employment of security men, erection of perimeter fences and availability of accommodation for health workers in the facility premises **(C)**, then health workers are likely to feel safer and therefore willing to work (i.e. provide MCH services) during night hours **(M)**, thus ensuring the provision of round the clock MCH services, and improved access to MCH services **(O)**

PPT4: Implications of withdrawal of support

In a context where basic support to health facilities (e.g. staff salaries, electricity, equipment and supplies etc.) is dependent on project funding, a sudden withdrawal of political and financial support to previously-funded MCH programme will limit availability of human and material resources **(C)**, making health workers feel unappreciated and unsupported (M), resulting in low morale and distrust among health workers and reduced performance, which can ultimately constrain the sustained provision of MCH services **(O)**.

BOX 2: Demand side programme theories

INITIAL WORKING THEORY (IWT2): contained in Phase 1 Methods Handbook
If poorly-functioning and inadequately-remunerated WDCs who are unaware of available MCH services in Anambra State, are mobilized, trained and financially incentivized in a timely manner **(C)**, these interventions will inspire WDC confidence and ownership of community health services **(M)** leading to increased and sustained identification and referral of pregnant women and increased utilization of MCH services **(O).**
**APPROVED PROGRAMME THEORIES—DEMAND**
**PPT1:Supporting women to access health care**<br>In the context of pregnant women's inability to pay for transportation to health facilities, or for medicines and MCH services, if WDCs are mobilized and trained; and pregnant women are provided CCT; and if VHWs encourage and support women to attend MCH services **(C),** then pregnant women will feel safer and confident to regularly attend health facilities **(M),** thus leading to increased and sustained utilization of health facility-based MCH services (such as ANC, deliveries and postnatal care), and ultimately to better MCH service outcomes **(O).**
**PPT2: Motivated and better performing VHWs**<br>In the context where pregnant women are financially-incentivised to access MCH care, if village health workers are provided with CCT and means of transportation to enable them mobilise communities and support women to reach hospitals **(C)**, these VHWs will feel more recognised by communities and motivated to encourage and accompany pregnant women to facilities for MCH services **(M)** thus contributing towards increased and sustained utilisation of MCH services by the pregnant women **(O)**.
**PPT3: Distrust due to withdrawal of incentives**<br>In the context of on-going targeted programme to improve access to MCH services to vulnerable pregnant women from remote rural areas, the sudden withdrawal of monetary and non-monetary incentives to support pregnant women to attend the continuum of MCH care **(C),** will help generate distrust from these women of health workers and wider system, and demotivate pregnant women from attending health facilities **(M),** eventually leading to reduced utilization of available facility-based MCH services **(O).**
**PPT4: Sustainability of trust in the health system**<br>In the context of improved staff attitude, upgraded health facilities and functioning WDCs achieved during implementation of the SURE-P programme, pregnant women who receive sustained financial and non-financial incentives to use MCH services **(C)**, are likely to develop and maintain a sense of improved trust (including confidence and satisfaction) with health facilities and staff **(M)**, ultimately leading to improved likelihood of repeated and regular utilisation of MCH services from these health facilities **(O)**.
