# Supplementary material for: Which mechanisms explain the motivation of primary health workers? Insights from the realist evaluation of a maternal and child health programme in Nigeria
Source: BMJ Glob Health. 2020 Aug 25;5(8):e002408. doi: 10.1136/bmjgh-2020-002408 (PMC7449364; doi:10.1136/bmjgh-2020-002408)
Supplement: Supplementary data [file bmjgh-2020-002408supp002.pdf]

Supplementary Material 2: Generic guide for In-depth Interviews

PROJECT PHASE 2

This generic guide should be customized for the following sub-groups of respondents and roles of specific individuals in relation to SURE-P/MCH care as perceived by the researchers:

Policymakers; Heads of facilities; Health workers; Service users and Community representatives (e.g. WDC reps)

Guidance notes

Before the interview

- Specify how long IDIs will take. You should familiarize yourself with the question guide and mark the priority questions for the interview on the interview guide accordingly.
- You should also be familiar with the definitions and the terminology used in the REVAMP glossary. You should be ready to clarify certain concepts and what they mean to the respondents during the interview
- As the interviews will be audio-recorded, you need to check that the recorder is working properly before going into the interview.
- You will also need paper notebooks and pens for note-taking and/or laptop computers as appropriate.
- Prepare extra copies of the project flyer, informed consent agreement and other key documents that are deemed useful for each interview.

At the beginning of an interview

- Introduce yourself and other researchers with you (if applicable).
- Briefly explain the objectives of the research and obtain informed consent.
- Check the overall time available for the interview.
- Explain that this will be an open interview and make sure that the respondent understands what is expected from her/him e.g.

"In your own words, we would like you to tell us your personal knowledge and experiences of providing/making use of mother and child health services in Anambra State."

During the interview

- Remember, although we are interested in the respondent's own knowledge and experiences, for the purpose of RE, you are allowed to tactfully interrupt respondents to clarify issues related to the initial working theories or provide prompts. But do not try to always fill the silence with a next question or probe—your respondent may need some time to think about the issue.
- The bullet points in the next page provide a checklist of possible issues to raise. Use your judgement and ask these bullet points only if the conversation is faltering, or if the respondent is deviating too much from the case study. Please note that some bullet points may not be applicable to some cases.
- Keep track of the questions asked;
- Keep track of any important emergent issues the interviewer should ask a follow-up question on, and remind the interviewer near the end of the interview
- Take note of any non-verbal clues that might be important for analysis.

- If the interview is not being recorded (due to respondent refusal, or equipment failure), the note-taker's priority is to summarise the content of the interview
- During the interview, the interviewer should listen and be prepared to ask follow-up, further clarifying and probing questions
- Use probes carefully; you do not need to ask all probes for all questions. Use your judgement and apply probes when the respondent is not answering fully or freely, or if the respondent doesn't appear to understand the question.
- Probe for specific examples from respondent's experience in support of her/his statements where possible.
- If the respondent is not answering a question in detail, ask 'give-me-an-example' type question to complement the question route.
- Always let the respondent finish a phrase; try not to interrupt her/him
- Use the interview guide flexibly, allowing the respondent to address the questions in any order but ensuring that all the key issues are covered. You may like to use the 'question asked?' column in the interview guide to keep track of which questions have been covered.

After the interview

- Thank the respondent/interviewee
- Remember to catalogue the tape/file (if you are recording)
- During debriefing, reflect on, discuss in the research team and clearly note down any other issues (e.g. respondent's mood during the pauses, your general impressions of the interview, good questions to ask) that may be important for future interviews, and the data analysis.
- Transcription if possible should start the day of the interview or at most the next day.

Sample questions and prompts for the sub-theories and logic behind the questions. Please note that these are illustrative questions to give you an idea of how specific IWTs can be explored. Please use your judgement during the actual interviews to explore the theory(ies) of most relevance to the particular respondent.

Note: Questions related to supply side sub-theories (in green rows) are suitable for health workers and policymakers.

**Example questions and prompts for supply side theories**	**Logic behind the questions**
Introduction by respondent	
For the tape, can you introduce yourself, and describe your work/what you do?	This question is to get respondents talking
**Prioritisation of MCH**<br>In the context of poor health outcomes, interest from policymakers and politicians in maternal and child health care (MCH), combined with advocacy and lobbying from key policy actors to prioritise MCH **(C)**, is likely to help generate and maintain political and economic commitment across all tiers of government manifesting as a culture of ownership of MCH programs **(M)**, eventually leading to timely release of counterpart funds and availability of other resources (e.g. human, supplies), ultimately contributing to sustained implementation of and access to MCH services for vulnerable groups **(O)**.	

**Example questions and prompts for supply side theories**	**Logic behind the questions**
**Broader questions to establish/confirm the key 'facts' about sub-theory**<br>• What is the current Government interest and commitment in MCH?<br>• Who are the key advocates/lobbyists for MCH?<br>• Which specific events are you aware of?	To get respondent s talking about insight related to the information needs matrix
1) Literature suggests that when the head of state is interested in a programme, all tiers of government (federal, state and LGA) will be commitment to that programme. How does this compare with your experience of SURE-P programme?<br>**Prompt:** Clarify interest of heads of state in the SURE-P programme and effect on Govt commitment on the programme	Questions 1-2 are introductory, to get respondents talking. They also check individual context.
2) Our discussions with researchers, suggest that advocating to and lobbying Govt to give priority to a programme (e.g. SURE-P) can increase political and economic commitment that programme. In your own experience, are you aware of advocacy and lobbying efforts for Govt. to prioritise the SURE-P programme?<br>Prompt:<br>• Which advocacy and lobbying efforts are you aware of, by who and the effects of the efforts?	Questions 1-2 are introductory, to get respondents talking. They also check context.
3) Some health workers suggest that when the head of state and all tiers of government are committed to a programme, this can stimulate timely release of funds and availability of human and other resources in the programme. Does this reflect your experience of the SURE-P programme in Anambra state?<br>Prompt:<br>• Clarify examples of government commitment to SURE-P and effect on timely release of funds and available resources<br>• Similarly ask for instances of non-commitment to SURE-P and why? Also ask observable effects on service delivery.	Checking mechanism and link between Govt commitment and timely release of funds/other resources.
4) Reports suggest that timely release of counterpart funds and availability of other resources leads to sustained implementation and access to MCH services pregnant women. Has this been your experience of SURE-P programme in Anambra State?<br>Prompt:<br>• Clarify examples of timely release of funds and availability and their effects on service delivery	Looking for outcome and links between timely release of funds and sustained implementation of and increased access to MCH
**Improved staff motivation**<br>In the context of staff shortages and lack of material resources, if adequate numbers and mix of skilled health workers are recruited, deployed to health facilities (that have security men, comfortable accommodation, regular electricity and water supply and transportation for emergency referrals); and if health workers receive adequate equipment, supplies and consumables for their work and are regularly trained, supervised and rewarded for good performance **(C)**, then health staff will feel motivated (i.e. appreciated and happy) to increase and maintain their performance **(M)** which is likely to lead to increased provision and utilisation of quality MCH services, ultimately contributing to improved service and health outcomes **(O)**	

Example questions and prompts for supply side theories	Logic behind the questions
**Broader questions to establish/confirm common starting point about sub-theory**<br>• What factors influence staff motivation?<br>• What does staff performance mean?<br>• What factors determine staff performance? How are they different from determinants of staff motivation?	To get respondent s talking about insight related to the information needs matrix
1) Some nurses say that adequate numbers and categories of staff and the availability of equipment and supplies, make staff feel appreciated and happy to increase their performance. Does this resonate with your experience of working in this facility?<br>Prompts:<br>• Clarify adequacy of numbers/mix of staff in facility and any change in numbers and mix over time?<br>• Probe effect of availability of staff and other resources on staff motivation to work	Checking for context (availability of staff, equipment and supplies in a facility)
2) The literature suggests that regular training, supervision and rewarding staff for good performance can motivate staff to increase/maintain their performance. Is this your own experience of determinants of increased staff motivation?<br>• If yes, probe for specific examples of how these factors led to improved performance<br>• Also probe for any other key factors affecting staff performance and why<br>• Clarify regularity of staff training, supervision and rewards?	Checking context of training, supervision and rewards in facility
3) Discussions with your colleagues suggest that staff who are motivated are likely to increase and maintain their performance. How does this compare with your own experience?<br>Prompts:<br>• Ask for instances of where staff motivation leading to increased performance	Checking for outcome (i.e. increase and sustainability of performance) and the link between motivation and staff performance
4) Reports suggest that sustained staff performance leads to improved delivery and utilisation of services. Does this represent your own experience in this UHC?<br>Prompts:<br>• Probe for specific example(s), and how performance affected service delivery and utilisation	Checking for outcome
5) Our reading of evaluations from other countries show that consistently improved delivery and utilisation of services result in better health outcomes. How does this compare to the situation in this facility?<br>Prompts:<br>• Probe for example(s), and how delivery and utilisation led to better health outcomes in relation to SURE-P programme	Checking for outcome and for link between service delivery and better health outcomes
**PPT3: Health workers feeling safer to work (S3)**<br>In the context where health facilities or communities ensure employment of security men, erection of perimeter fences and availability of accommodation for health workers in the facility premises **(C)**, then health workers are	

**Example questions and prompts for supply side theories**	**Logic behind the questions**
likely to feel safer and therefore willing to work (i.e. provide MCH services) during night hours **(M)**, thus ensuring the provision of round the clock MCH services, and improved access to MCH services **(O)**	
**Broader questions to establish/confirm the common starting point about sub-theory**<br>• How secure do you feel in your facility and why?<br>• What key issues do you think can ensure provision of round the clock services, and why?	To get respondent s talking about insight related to the information needs matrix
1) Our analysis suggests that suggest that a secure hospital compound and availability of staff accommodation increases staff safety and preparedness to work at odd hours? I wonder what do you think about this?<br>Prompts:<br>• Can you think of an example where lack of safety has acted as a barrier to motivation/commitment? Or the other way round, when the security issue was sorted, staff behaved differently?	Checking for meso context and link between security and motivation to work odd hours
2) Some staff report that assurance of a secure facility leads to the provision of 24Hr health services. What is your own experience of this?<br>Prompt:<br>• Probe for evidence of a secure facility and examples of how this resulted to round the clock service delivery	Looking for context and mechanisms
3) Data analyses suggest that provision of round the clock services improve access to MCH services by pregnant women. How do you think these two factors ate interrelated??<br>Prompt:<br>• What do you think the reward DO to increase motivation?<br>• Can you think of examples when women were getting rewards but still were not motivated to come to hospitals?<br>• What do you think the barriers were there?	Looking for mechanisms and outcomes
**PPT2: VHW motivation for better performance**<br>In the context where pregnant women are financially-incentivised to access MCH care, if village health workers are provided with CCT and means of transportation to enable them mobilise communities and support women to reach hospitals **(C)**, these VHWs will feel more recognised by communities and motivated to encourage and accompany pregnant women to facilities for MCH services **(M)** thus contributing towards increased and sustained utilisation of MCH services by the pregnant women **(O)**	
**Broader questions to establish/confirm common starting point about sub-theory**<br>•<br>•	To get respondent s talking about insight related to the information needs matrix
1) Discussion with midwives in other LGAs indicate that VHWs feel motivated and recognised when they receive financial rewards (CCT) and transport to mobilise/accompany women to hospital? Why do you think this is the case?<br>**Prompts:**	Checking for context and links between incentives and VHW motivation

**Example questions and prompts for supply side theories**	**Logic behind the questions**
• What do you think the reward DO to increase motivation?<br>• Can you think of examples when women were getting rewards but still were not motivated to come to hospitals?<br>• What do you think the barriers were there?	
2) The literature suggests that VHWs mobilising and accompanying women to hospital, leads to increased and sustained utilization of hospital services? Why is that the case?<br>**Prompts:**<br>- Probe how mobilising communities help? What is that VHWs do when they accompany women that helps? Is it just transport or do they have other roles (advocating, protecting, etc)?<br>- Probe evidence of increased utilization for services following VHW activities (MCH registers and VHW CCT registers). Any difference with other localities? Why?	Checking for outcome and links between VHW activities and increased utilization of services
3) Records show that giving pregnant women financial incentives to access MCH services, increases and sustains utilization of hospital services? How does this happen? How does financial incentives influence women's behaviour?<br>**Prompts:**<br>- Probe evidence of increased utilization for services following payment of CCT to women (MCH registers and CCT registers). Any difference with other localities? Why?	Checking for outcome
4) Our analyses show that some women did not receive CCT although they attended antenatal classes, delivered in hospital and attended postnatal care. Why do you think this was the case?	Checking for context
5) Analyses of transcripts also highlight some women receive a single bulk CCT at after they delivered at a health facility, whereas other women were paid CCT in instalments following their use of ANC, delivery and postnatal care respectively? Why do you think this was the case?	Checking for context

Note: The following questions related to supply side sub-theories (in green row) are suitable for health workers and policymakers.

**Example questions and prompts for supply side theories**	**Logic behind the questions**
Introduction by respondent	
For the tape, can you introduce yourself, and describe your work/what you do?	This question is to get respondents talking
**Supporting women to access health care**	

**Example questions and prompts for supply side theories**	**Logic behind the questions**
In the context of pregnant women's inability to pay for transportation to health facilities, or for medicines and MCH services, if WDCs are mobilized and trained; and pregnant women are provided CCT; and if VHWs encourage and support women to attend MCH services **(C)**, then pregnant women will feel safer and confident to regularly attend health facilities **(M)**, thus leading to increased and sustained utilization of health facility-based MCH services (such as ANC, deliveries and postnatal care), and ultimately to better MCH service outcomes **(O)**.	
**Broader questions to establish/confirm common starting point about sub-theory**<br>• What facilitates you/women use the MCH services and<br>• What prohibits, and why?<br>• What makes you feel safer to use services?<br>• What makes you feel more confident to use services?	To get respondent s talking about insight related to the information needs matrix
1) Discussion with community members suggest that pregnant women feel more confident to access health services when they receive monetary incentives to attend hospital services. How does this compare to your experience of the situation in this locality?<br>**Prompts:**<br>- Probe for types of and availability financial incentives for supporting pregnant women (CCT registers). Any difference with other localities? Why?<br>- probe link between incentives and women's confidence to use services	Questions 1-2 are introductory, to get respondents talking.<br><br>They also check context. (availability of financial and non-financial incentives).
2) Other evaluations suggest that being supported by VHWs to access services increases their confidence and persuasion to attend health facilities. Does this resonate with your knowledge of women's perception of attending facilities in this locality?<br>Prompt:<br>• Clarify health worker's awareness of women's perception of attending facilities unsupported by VHWs?	Questions 1-2 are introductory, to get respondents talking. They also check context.
3) Some workers suggest that who have positive relationships with VHWs, use health facilities more regularly than other women. Does this reflect your experience of women's utilization of facilities during the SURE-P programme? How is this different from other facilities?	Checking mechanism and link between VHW support and utilization of services.
**Sustainability of trust in the health system**<br>In the context of improved staff attitude, upgraded health facilities and functioning WDCs achieved during implementation of the SURE-P programme, pregnant women who receive sustained financial and non-financial incentives to use MCH services **(C)**, are likely to develop and maintain a sense of improved trust (including confidence and satisfaction) with health facilities and staff **(M)**, ultimately leading to improved likelihood of repeated and regular utilisation of MCH services from these health facilities **(O)**	
**Broader questions to establish/confirm common starting point about sub-theory**<br>• What is the role of WDCs in the SURE-P programme?<br>• How can WDCs become more functional for the benefit of pregnant women?<br>• What does trust in the health system means?	To get respondent s talking about insight related to the information needs matrix

**Example questions and prompts for supply side theories**	**Logic behind the questions**
1) Literature show that continuous and regular availability of incentives to pregnant women increases their trust in the health system. Is this your observation?<br>Prompts:<br>• Clarify availability and continuity of incentives to pregnant women<br>• Probe effectiveness of sustained incentives on women's trust for health system	Checking for context and mechanism
2) Discussions with pregnant women show that apart from monetary incentives, improved staff attitudes increased women's trust in health system. From your experience, what type of attitudes will increase the trust of women? And why?	Checking for mechanism
3) Discussion with pregnant women show that those who trust the health system are also likely to use health facilities repeatedly and regularly. Can we ask, how can we build trust in the health system?<br>• Probe for specific examples of how trust leads to repeated use of facilities	Checking outcome; and link between trust and repeated use of facilities
**Implications of withdrawal of support**<br>In a context where basic support to health facilities (e.g. staff salaries, electricity, equipment and supplies etc.) is dependent on project funding, a sudden withdrawal of political and financial support to previously-funded MCH programme will limit availability of human and material resources **(C)**, making health workers feel unappreciated and unsupported **(M)**, resulting in low morale and distrust among health workers and reduced performance, which can ultimately constrain the sustained provision of MCH services **(O)**	
**Broader questions to establish/confirm common starting point about sub-theory**<br>• Are you aware withdrawal of political/financial support to SURE-P programme?<br>• What factors affect staff morale in Anambra state?<br>• How did this affect service delivery?	To get respondent s talking about insight related to the information needs matrix
1) A common pattern in interviews with different staff groups is that nurses and midwives who work in health facilities previously supported by SURE-P programme feel demoralized after suspension of funds to the SURE-P? What led to withdrawal of political and financial support to SURE-P? Why do you think it affected staff morale so badly<br>**Prompts:**<br>• Probe current situation of human and material resources in health facility? And its effect on staff morale	Checking for context
2) We find from our analysis that health workers who are demotivated (unappreciated) also distrust government and are unhappy with health system. How does low staff morale affect trust in this facility?	Checking for outcome
3) We find from speaking with nurses that low staff morale and distrust in the government or health system, also affects staff performance. Why do you think this is the case? How do morale and trust affect staff performance?<br>**Prompt:**<br>Probe for specific examples of how morale and trust improved performance	Checking for outcome

**Example questions and prompts for supply side theories**	**Logic behind the questions**
4) Analysis of data show that prolonged reduction in individual staff performance can affect the overall quality of services provided in a health facility. What is your observation of the link between individual staff performance and the health facility's quality of services?<br>**Prompts:**<br>• Probe for examples of how staff performance improved quality of service.<br>• Are there times when increased staff performance didn't lead to better quality of service? What was the barrier here?	Checking for outcome
**PPT3: Distrust due to withdrawal of incentives**<br>In the context of on-going targeted programme to improve access to MCH services to vulnerable pregnant women from remote rural areas, the sudden withdrawal of monetary and non-monetary incentives to support pregnant women to attend the continuum of MCH care (C), will help generate distrust from these women to health workers and wider system, and demotivate pregnant women from attending health facilities (M), eventually leading to reduced utilization of available facility-based MCH services (O)	
**Broader questions to establish/confirm common starting point about sub-theory**<br>• What factors promote women's attendance of MCH services and what prohibits, and why?<br>• What factors affect women's trust in health workers and health system in general?	To get respondent s talking about insight related to the information needs matrix
1) Discussions with pregnant women suggest that withdrawing monetary and non-monetary support from pregnant women make them lose faith in health workers and the health system. What do you know of suspension of incentives to pregnant women in this community?<br>Prompts:<br>• What does trust in health workers mean for pregnant women? Also check meaning of trust in health system<br>• Probe effects of suspension of incentives on trust?<br>• Are there instances where suspension of incentives did not affect trust? Why?	Checking for context<br><br>Similar to question on sustainability of trust in health system
2) We find from our analysis that pregnant women who distrust health workers and the wider health system stop attending the health facilities. In your opinion How does trust affect women's attendance in this facility?<br>**Prompt:**<br>• Are there times when attendance decreased even when trust in health workers was high? What was the barrier in that example?	Checking outcome
3) Reports from countries show that when many women stop attending hospitals, the overall utilization of services in the facility reduces. How does this information compare with utilization of services in this health facility?<br>**Prompt:**<br>• Probe examples of how non- and poor attendance affected overall service utilization in the facility<br>• Check utilization register to see changes in service utilization	Checking outcome
